# Editorial: Women in hepatology: 2023

**DOI:** 10.3389/fgstr.2024.1374777

**Published:** 2024-05-07

**Authors:** Atoosa Rabiee

**Affiliations:** Washington DC VA Medical Center, United States Department of Veterans Affairs, Washington, DC, United States

**Keywords:** women, hepatology, liver, research, gender

There is no shortage of outstanding women in hepatology who have dedicated their lives and careers to research, teaching, clinical care, program development, and chairing as presidents of national societies.

Progress has been slow but steady. From Dr. Sheila Sherlock, who pioneered the Journal of Hepatology as one of its founding editors in 1976, to the first president of the American Association for Study of Liver disease (AASLD), Dr. Teresa Wright, in 2005, this trend was followed by Dr. Guadalupe Garcia-Tsao as the first Hispanic woman president of AASLD in 2012 and Dr Anna Lok as the first Asian American president of AASLD in 2017. Dr. Laurie DeLeve and Dr. Norah Terrault were subsequently elected as AASLD presidents in 2022 and 2023, respectively (1).

Women make up approximately half of the hepatology workforce (2).

Current society membership and the percentage of hepatology fellowship applicants also support similar numbers. 

However, equity in compensation and leadership remains a major issue despite the contributions of many strong female hepatologists to this field. A recent survey, showed that despite their role in education, women are less likely to get protected time to fulfill these duties (3). This survey also noted higher base compensation for men compared with women in the field of liver transplant which resulted in 8% less income compared to men.

More recently, many societies have brought attention to equity and inclusion specifically highlighting women in hepatology.

Dedicating a Research Topic of Frontiers in Gastroenterology to research conducted and published by women in hepatology is certainly a step in the right direction and a strategy to promote equity that we hope will be more widely adopted by other journals and societies.

This and other efforts encourage women to support one another and provide opportunities for professional growth. 

The first step in solving an issue is to acknowledge it exists. If we acknowledge that the field is not leveled and has not been for many years, and if we accept that there are many talented women in the field who have not received recognition due to inequities, we can start by brainstorming how to solve the problem.

Arguably, as part of the few medical fields established by a woman, namely, Dr. Sherlock, we have to commit to addressing gender inequity in all aspects of women’s careers.

As Dr. Guadalupe Garcia-Tsao said “it is our collective job to empower all women” (4).

And we salute Frontiers for taking a step in the right direction.

## Author contributions

AR: Writing – original draft, Writing – review & editing.
